# *Meloidogyne incognita* management by nematicides in tomato production

**DOI:** 10.21307/jofnem-2021-055

**Published:** 2021-07-19

**Authors:** Zane J. Grabau, Chang Liu, Rebeca Sandoval-Ruiz

**Affiliations:** 1 Entomology and Nematology Department, University of Florida 1881 Natural Area Drive, Gainesville, FL 32611

**Keywords:** 1,3-dichloropropene, Fluopyram, Management, *Meloidogyne incognita*, Nematicide, Fumigation, Nematode community, Oxamyl, *Solanum lycopersicum*, Southern root-knot nematode, Tomato

## Abstract

*Meloidogyne incognita* (southern root-knot nematode, SRKN) is a major pest in tomato (*Solanum lycopersicum*) production in the Southeastern United States. Management has relied on fumigant and carbamate non-fumigant nematicides. New non-fumigant nematicides, such as fluopyram, are available and field evaluation of new nematicides is needed. The objectives of this research were to assess the efficacy of new (fluopyram) and established (oxamyl) non-fumigant nematicides as well as fumigation (1,3-dichloropropene) for (1) SRKN management, and (2) impacts on total soil abundances of non-target, free-living nematodes in field tests in Florida. Fumigation with 1,3-D consistently managed SRKN and, in two of three trials, increased yield relative to untreated. Oxamyl and fluopyram also had efficacy in managing SRKN, but were inconsistent from year to year. Oxamyl provided better root galling control than fluopyram in one of two trials, but otherwise those nematicides provided similar SRKN management and yield response. Supplementing 1,3-D fumigation with fluopyram did not improve SRKN management or yield relative to fumigation alone. Fumigation consistently reduced free-living nematode abundances relative to untreated. Oxamyl and fluopyram were more inconsistent, but always reduced total free-living nematode abundances when effective against SRKN. In summary, while non-fumigant nematicides provided some management of SRKN, fumigation continued to be the most consistent option. All nematicides had deleterious effects on free-living nematodes.

The production of tomato (*Solanum lycopersicum*) is a very important industry in the United States with 10 billion kg tomatoes worth $1.6 billion United States dollars (USD) produced in 2019 (USDA-NASS, 2020). Florida produces 54% of fresh market tomatoes, an industry that produced 646 million kg worth $705 million (USD) nationwide in 2019 (USDA-NASS, 2020). *Meloidogyne incognita* (southern root-knot nematode, SRKN) is a major pest in tomato production, and there are relatively few management options available (Desaeger et al., 2017; Koenning et al., 1999; Regmi and Desaeger, 2020). Crop rotation has a limited role in managing SRKN in tomato production because most economically viable rotation cash crops are also hosts of SRKN (Anwar and McKenry, 2010; Hajihassani et al., 2019). While certain cover crops can help manage SRKN (Kokalis-Burelle et al., 2013; Wang et al., 2008), it is generally not economically viable to deploy them for a long enough period to adequately manage SRKN on their own. Tomato cultivars resistant to SRKN are available, but adoption is limited due to a variety of factors (Regmi and Desaeger, 2020). Therefore, SRKN management relies heavily on fumigation, with 1,3-dichloropropene one of the most common chemistries used for nematode management in the Southeast. Older non-fumigant nematicides have also been commonly used to supplement fumigation or in cases when fumigation is not allowed, such as double-cropping on perforated plastic mulch. Regulatory restrictions have left oxamyl, a carbamate, as the remaining non-fumigant nematicide among older chemistry classes still labelled for tomato production in the United States.

Because of the reliance on a small selection of older nematicides, the development of new tools for SRKN management is important. One group of new tools are the benzamide, non-fumigant nematicides, such as fluopyram. Fluopyram is a succinate dehydrogenase inhibitor (SDHI) that was first used as a fungicide (Cordova et al., 2017; Kandel et al., 2019). There is a growing body of knowledge about fluopyram efficacy against SRKN in tomato or vegetables, although more field efficacy data are needed for robust evaluation. Fluopyram has nematicidal or nematistatic activity against SRKN in vitro (Faske and Hurd, 2015; Ji et al., 2019; Oka and Saroya, 2019). Similarly, in greenhouse and microplot tests with tomato or cucumber (*Cucumis sativus*), fluopyram typically helps manage SRKN (Dahlin et al., 2019; Nikoletta et al., 2019), although performance relative to other non-fumigant nematicides has varied from somewhat worse (Jones et al., 2017) to comparable (Silva et al., 2019) to somewhat better (Hajihassani et al., 2019; Li et al., 2020; Yue et al., 2020).

There have been few field tests of fluopyram against SRKN. Fluopyram compared favorably to abamectin for SRKN management in field tomato production (Ji et al., 2019), but did not provide adequate coverage depth in the soil profile for carrot (*Daucus carota*) production (Becker et al., 2019). Fluopyram efficacy against other nematodes in field vegetable research has also varied, as fluopyram suppressed *Belonolaimus longicaudatus* but not *Meloidogyne hapla* in strawberry (*Fragaria* x *ananassa*) (Watson and Desaeger, 2019), was inconsistent against *Meloidogyne javanica* in tomato (Desaeger and Watson, 2019), and was inconsistent against *M. javanica*, *M. hapla*, and *B. longicaudatus* in various vegetables (Khanal and Desaeger, 2020). Likewise in peanut (*Arachis hypogea*) or cotton (*Gossypium hirsutum*) as an in-furrow application, fluopyram was inconsistent against *Meloidogyne arenaria* (Grabau et al., 2020), and either inconsistent (Grabau et al., 2021) or ineffective against *Rotylenchulus reniformis* (Schumacher et al., 2020). Nematicides are often less effective in field than lab or greenhouse settings, and field testing is particularly important for fluopyram because it is poorly mobile (Faske and Brown, 2019). Limited mobility may reduce coverage area, which is important in field production, but may not be apparent in confined greenhouse or lab tests. In addition, field evaluation of SRKN management by fluopyram relative to fumigants and carbamate nematicides is needed, as these products serve as a historical standard.

In addition to nematicide efficacy against target plant-parasitic nematodes, minimizing impacts on non-target organisms, such as free-living nematodes, is increasingly valued. Free-living nematodes may contribute to productive soil by providing services such as nutrient cycling (Holajjer et al., 2016; Trap et al., 2016), pathogen management (Khan and Kim, 2005), and microbe redistribution (Jiang et al., 2018). Free-living nematodes also serve as a proxy for the soil community at large, since they are involved at multiple trophic levels (Ferris et al., 2001; 2012).

Fluopyram, such as other benzamides, has a narrower toxicity range than fumigants or organophosphate and carbamate nematicides against higher organisms (ESFA, 2013), but, in the few studies conducted, fluopyram has had mixed impacts on free-living nematodes. In turfgrass field studies, fluopyram had more non-target effects on free-living nematodes than other non-fumigant nematicides (Waldo et al., 2019), whereas fluopyram had non-target effects on bacteria-feeding, but not fungal-feeding nematodes in a tomato greenhouse assay (Nikoletta et al., 2019). In peanut and strawberry production, fluopyram had no or minimal impact on free-living nematodes (Desaeger and Watson, 2019; Grabau et al., 2020). Application rates and methods vary by crop, which may account in part for differences across systems and make it important to investigate specific crops, such as tomato production. In contrast, 1,3-D typically has broad spectrum effects against a range of free-living nematodes (Sanchez-Moreno et al., 2010; Timper et al., 2012; Watson and Desaeger, 2019). Despite its long history, the impact of oxamyl on free-living nematodes is not well-described. In tomato microcosm (Carrascosa et al., 2015) and field experiments (Ntalli et al., 2018), oxamyl did tend to decrease free-living nematode abundances relative to untreated. Field testing on non-target effects of these nematicides, relative to each other, in tomato production would aid growers in deciding what chemistries to deploy, particularly when target efficacy of nematicides under consideration is similar.

Based on these needs, the objectives of this research were to assess fluopyram, oxamyl, and 1,3-D for (1) SRKN management, and (2) impacts on total soil abundances of non-target, free-living nematodes.

## Materials and methods

### Experimental design

To test these objectives, three field trials (2018, 2019, and 2020) were conducted at the University of Florida North Florida Research and Education Center-Suwannee Valley located near Live Oak, FL (30°18′07.6″ N, 82°54′03.3″ W). The soil was a Chipley–Foxworth–Albany complex (91% sand-6.8% silt-2.4% clay; 1.6% organic matter).

A preliminary experiment to test the efficacy of nematicides against SRKN was conducted in 2018. The experiment was a randomized complete block design with five replicates. The nematicide treatments included 1,3-dichloropropene (1,3-D) and fluopyram combinations as described in Table 1. The 1,3-D was applied in the form of Telone II (Corteva Agrisciences, Wilmington, DE). The 1,3-D was applied to flat ground as a broadcast treatment 22 days before planting (DBP) using a shank fumigation rig. The rig was configured with five coulters to open traces immediately in front of the five shanks with press wheels behind each shank to seal traces. Shanks were spaced at 30–cm intervals for a total application band of 2.03 m, and nematicide was released at 25–cm deep in the soil profile.

**Table 1.** Nematicide treatments in 2018–2020 trials.

Treatment	Active ingredient	Product	Total product broadcast rate^a^	Total a.i. broadcast rate	Timing/method
	2018				
1	Untreated control				
2	1,3-dichloropropene (1,3-D)	Telone II	140 liters/ha	166 kg/ha	Preplant shank fumigation
3	1,3-D	Telone II	140 liters/ha	166 kg/ha	Preplant shank fumigation
	Fluopyram	Velum Prime	474 mL/ha	237 g/ha	Single chemigation 21 DAP
4	Fluopyram	Velum Prime	474 mL/ha	237 g/ha	Single chemigation at planting
5	Fluopyram	Velum Prime	474 mL/ha	237 g/ha	Chemigation (1) at planting and (2) 21 DAP
	2019 and 2020				
1	Untreated control				
2	1,3-D	Telone II	140 liters/ha	166 kg/ha	Preplant shank fumigation
3	1,3-D	Telone II	140 liters/ha	166 kg/ha	Preplant shank fumigation
	Fluopyram	Velum Prime	474 mL/ha	237 g/ha	Single chemigation at planting
4	Fluopyram	Velum Prime	474 mL/ha	237 g/ha	Single chemigation at planting
5	Oxamyl	Vydate L	3.10 liters/ha	750 g/ha	Chemigation (1) at planting and (2) 10-12 DAP

Note: ^a^Fluopyram and oxamyl were chemigated in a 0.61 m bed with 1.83 m row spacing, so rate per treated acre is three times that of the broadcast rate. For 1,3-D application, broadcast and treated rates are the same.

Fluopyram was applied as Velum Prime (Bayer Cropscience, Research Triangle Park, NC) by chemigation at transplanting or 21 days after planting (DAP) depending on treatment as described in Table 1. Fluopyram was chemigated through a single, surface drip tape per plot. Plots with the same treatment were plumbed through a common irrigation line. Irrigation was applied for 10 min before any nematicides were applied. Fluopyram was then applied through irrigation lines for approximately 10 min using a Masterflex L/S (Cole Parmer, Vernon Hills, IL) multi-line pump with one line per treatment. After nematicide injection, irrigation was applied for approximately 10 additional minutes to incorporate nematicide into the soil. Emitter spacing on drip tape was 30 cm. Total water volume per cycle was 41,316 liters/ha, based on application to only the 60-cm-wide beds. Treatments not scheduled to receive nematicide at a particular timing received irrigation but no nematicide. Soil temperatures at 10 cm below the soil surface were 30.6, 30.0, and 31.1°C at fumigation, transplanting, and post-plant chemigation, respectively.

The main experiment was conducted in 2019 and repeated in 2020. Treatments were 1,3-D and fluopyram, alone or in combination, as well as oxamyl alone, as described in Table 1. Oxamyl and fluopyram rates were based on the maximum labelled rate, whereas 1,3-D rate was based on local standard practices. The 1,3-D fumigation was conducted as described for the 2018 trial, except that fumigation was conducted 44 and 43 days before planting in 2019 and 2020, respectively. Fluopyram and oxamyl treatments were chemigated as described for fluopyram in the 2018 trial at the timings specified in Table 1. Oxamyl was applied in the form of Vydate L (Corteva Agrisciences, Wilmington, DE). Soil temperatures (10‒cm depth) in 2019 were 31.8, 29.9, and 32.1°C at fumigation, transplanting, and post-plant chemigation, respectively. Soil temperatures in 2020 were 32.0, 30.3, and 27.3°C, at fumigation, transplanting, and post-plant chemigation, respectively.

### Trial management

Timing of important trial management and data collection events are summarized in Table 2. In each trial, a plot consisted of 1 plasticulture tomato bed (60–cm wide) that was 12.2–m long with 1.83–m row spacing (center to center) and 3.05–m unplanted buffer lengthwise between plots. Approximately 10 days before planting, beds were shaped mechanically by pulling soil from a 1-m-wide band and formed with reflective low density polyethylene plastic mulch. Lengthwise and crosswise unbedded areas between bedded plots ensured fumigant did not move between beds. Trials were transplanted with root-knot nematode susceptible tomato (“Grand Marshall” in 2018 and “BHN 602” in 2019 and 2020) on August 23, 2018, September 5, 2019, and September 5, 2020. Plant spacing was 45 cm. Weed, fertility, disease, and insect management was uniform across the trial and done in accordance with local practices. Irrigation was regulated to maintain soil moisture at 8–12% volumetric water content using a time-domain reflectometry (TDR) soil moisture probe. Irrigation was applied daily, if needed, for 30 min.

**Table 2.** Schedule for data collection. Numbers in parentheses are days before transplanting (DBP) or days after transplanting (DAP).

Item	2018	2019	2020
Preplant soil samples	30 July (23 DBP)	30 July (44 DBP)	27 July (43 DBP)
Preplant *M. incognita* J2/100 cm^3^ soil	30	45	14
Soil fumigation	1 August (22 DBP)	30 July (44 DBP)	27 July (43 DBP)
Date planted	23 August	5 September	8 September
Tomato variety	Grand Marshall	BHN 602	BHN 602
Postplant fluopyam injection	13 September (21 DAP)	17 September (12 DAP)	18 September (10 DAP)
Harvest date(s)	9 November (78 DAP)	4 December (90 DAP)	2 December (87 DAP)
	20 November (89 DAP)		
	29 November (98 DAP)		
Soil sampling/root rating	29 November (98 DAP)	4 December (90 DAP)	3 December (88 DAP)

### Tomato yield

Tomatoes were harvested by hand in each trial. In 2018, all large-size tomatoes (greater than 6.99–cm diameter) were picked on November 9 (78 DAP) and November 20 (89 DAP). On November 29, 2018 (98 DAP) all tomatoes were picked, regardless of size, and the trial was terminated. Total yield and yield for each individual date were calculated for the 2018 trial.

For the 2019 and 2020 trials, tomatoes were harvested only once, due to slightly later planting, frost timing, and labor availability. Tomatoes were harvested on December 4, 2019 (90 DAP) and December 2, 2020 (88 DAP). In 2019 and 2020, tomatoes were mechanically sorted by size grade before weighing using a Kerian Speed Sizer (Kerian, Grafton, ND). Size classes were cull, small (6 × 7), medium (6 × 6), and large (5 × 6), representing diameters (cm) of < 5.7, 5.7 to 6.3, 6.4 to 7.0, and > 7.0, respectively. Size classes were based on the Florida Marketing Order (USDA-AMS, 2005). In addition to yield by size grade, marketable yield (small, medium, or large) and total yield (cull plus marketable) was calculated. For the 2019 and 2020 trials, basic economic data was calculated. Total crop value for each plot was determined based on reported price by size grade for the Central and South Florida shipping point at the time of harvest each year (USDA-AMS, 2019; USDA-AMS, 2020). In 2019, prices (in USD) were 11.95, 13.95, and $17.95 per 11.4 kg carton for small, medium, and large size grades, respectively. In 2020, prices were 17.95, 19.95, and $21.95 per 11.4 kg carton for small, medium, and large size grades, respectively. In addition, net income was calculated as crop value minus nematicide product cost. This did not consider labor, fuel, equipment, or other additional costs of nematicide application. Nematicide product costs were determined based on an informal survey of local agrochemical suppliers at the time of publication and are provided as an estimate only. Nematicide product cost (USD) estimates per hectare were $780 for 1,3-D alone; $851 for 1,3-D plus fluopyram; $72 for fluopyram alone; and $61 for oxamyl alone.

### Nematode quantification

Before each trial, a composition soil sample was collected from the trial site just before fumigation to verify *M. incognita* infestation. Using an Oakfield tube, 15 cores to 25–cm depth were collected randomly across the trial site and homogenized. Nematodes were extracted using the sucrose-centrifugation method (Jenkins, 1964), and quantified morphologically using a Primovert microscope (Carl Zeiss AG, Oberkochen, Germany) at 100 times magnification. There were 30, 45, and 14 *M. incognita* J2/100–cm^3^ soil before planting in 2018, 2019, and 2020, respectively.

For the 2019 and 2020 trials, at harvest, before tomato vines were terminated, soil samples were collected to determine *M. incognita* and free-living nematode abundances. From each plot, 12 soil cores to 25–cm depth were collected at the base of plants. Soil was mixed by hand, and nematodes were extracted as previously described. Both *M. incognita* J2 and total free-living nematodes abundances were quantified morphologically by microscope as previously described. Nematode soil abundances at harvest were not determine for the 2018 trial. For all three trials, root surface galling was quantified just after harvest. After harvesting, root surface galling was rated from 0 to 100% for 10 plants per plot (Barker et al., 1986).

### Statistical analysis

Each trial was analyzed separately. Data were subject to one-way ANOVA. Before completing ANOVA, response variables were transformed, if needed, to meet assumptions of homogeneity of variance using Levene’s test (Levene, 1960) and normality of residuals based on graphing (Cook and Weisburg, 1999). Specifically, total yield in 2018 and galling in 2020 were square-root transformed. Other variables were not transformed. For variables with significant (*α* = 0.05) treatment effects in ANOVA, means were separated by Fisher’s LSD (*α* = 0.05). Analyses were conducted in R statistical software (version 3.4.4, The R Foundation for Statistical Computing, Vienna, Austria).

## Results

### 2018 trial

**Figure 1:**
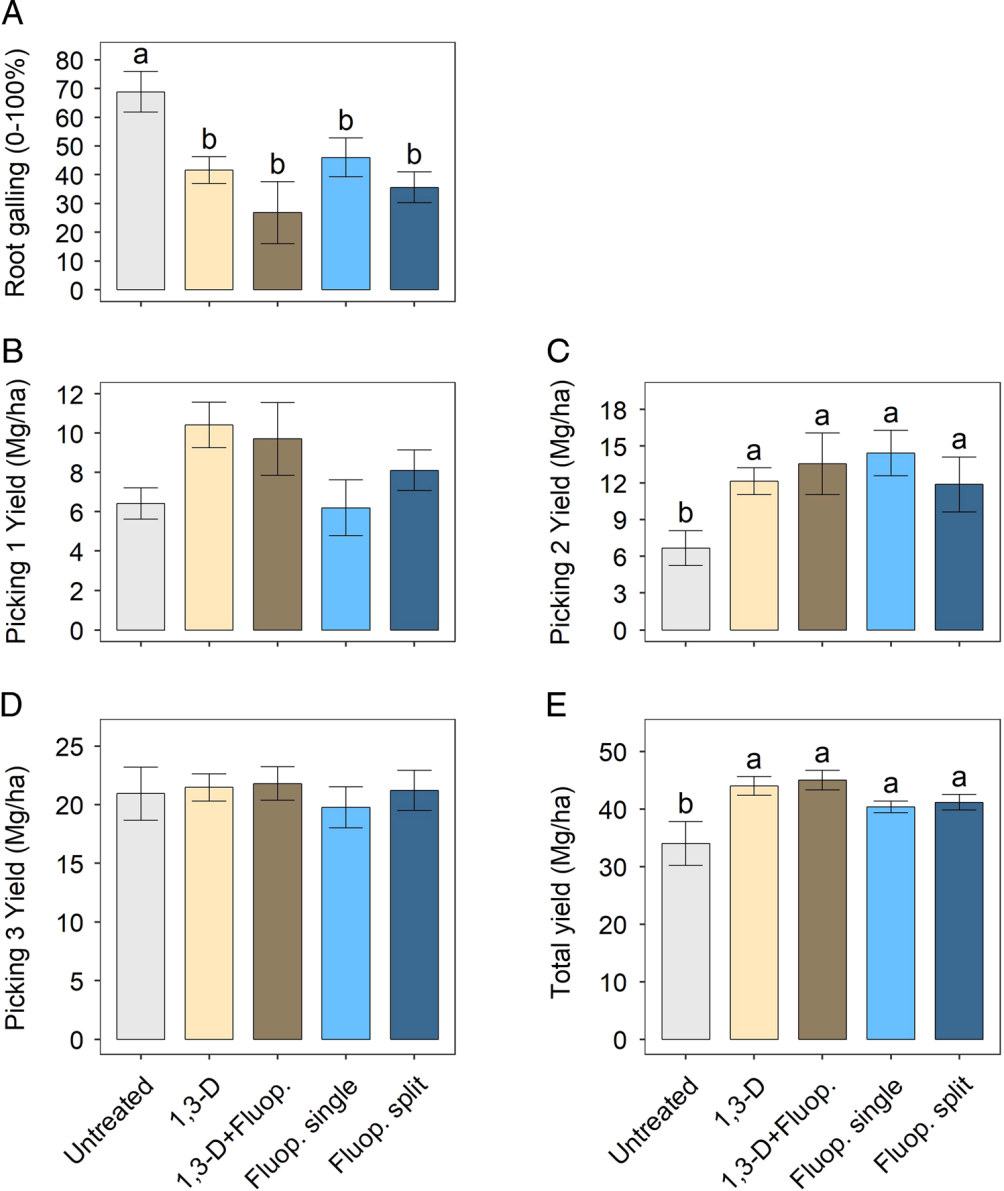
Nematicide effects on root galling at harvest (A) and tomato yields (B-E) in 2018 trial. Within each subfigure, means with different letters are significantly different (*P * < * *0.05) based on Fisher’s protected LSD. “1,3-D + Fluop.” indicates preplant 1,3-D followed by fluopyram (237 g a.i./ha) at 3 weeks after planting. “Fluop. single” indicates a single fluopyram chemigation (237 g a.i./ha) at transplanting. “Fluop. split” indicates split fluopyram chemigation with 118.5 g a.i./ha each applied at transplanting and 3 weeks after transplanting.

In the preliminary trial conducted in 2018, root galling at harvest was significantly lower with any nematicide treatment compared with untreated control (ranging from 33 to 61% reductions), but there were no significant differences among nematicide chemistries or combinations (Figure 1A). Similarly, total yield was greater for any nematicide treatment than untreated control (19–32% increases) with no significant differences among treatments with nematicide (Figure 1E). This was driven by yield at the 2nd picking, which had significant differences (Figure 1C), as there were no significant differences at the first (Figure 1B) or third picking (Figure 1D).

### Nematode management in 2019 and 2020 trials

**Figure 2:**
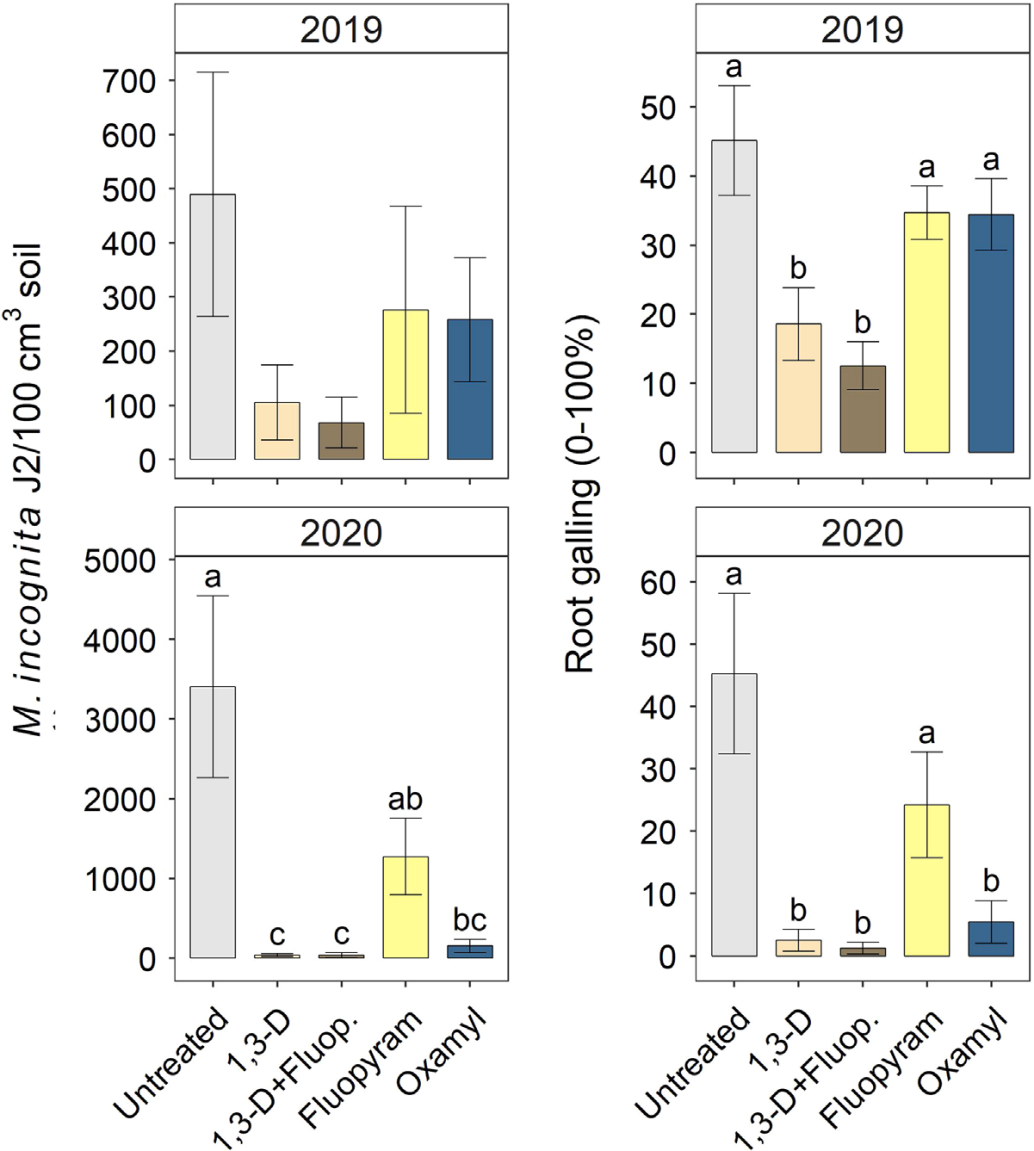
Nematicide effects on *Meloidogyne incognita* J2 soil abundances and root galling at harvest in 2019 and 2020 trials. Within each subfigure, means with different letters are significantly different (*P* < 0.05) based on Fisher’s protected LSD.

**Figure 3:**
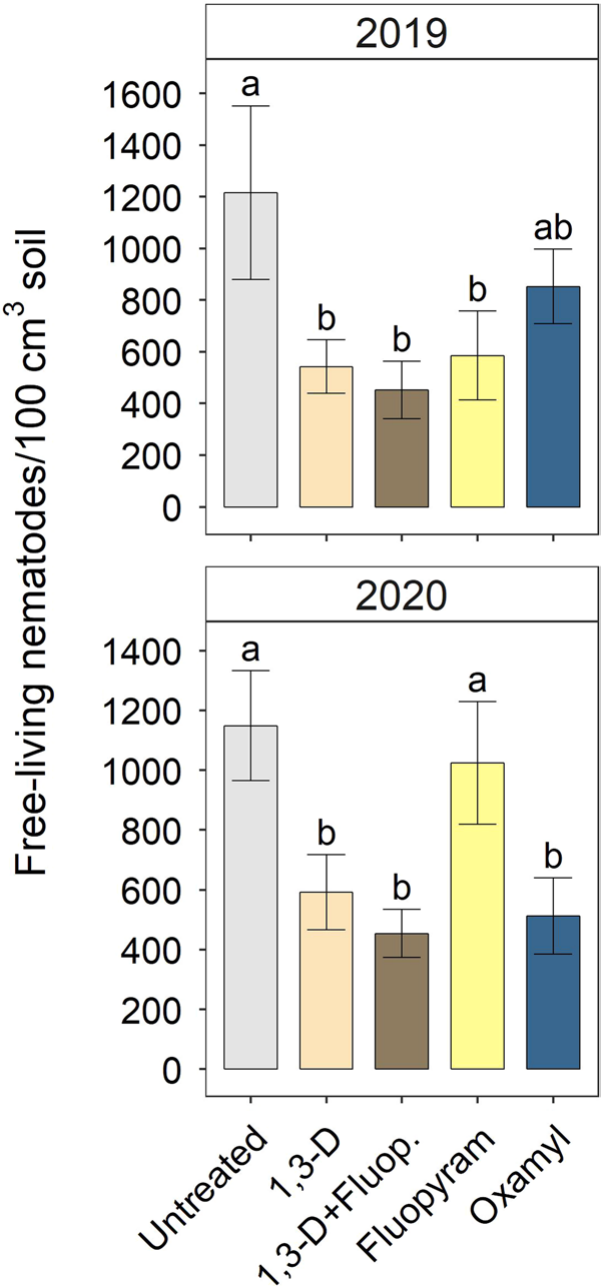
Nematicide effects on free-living nematode soil abundances in 2019 and 2020 trials. Within each subfigure, means with different letters are significantly different (*P * < * *0.05) based on Fisher’s protected LSD.

In the 2019 and 2020 trials, nematicides varied in their management of *M. incognita*. In 2019, treatments with 1,3-D alone or in combination with fluopyram reduced root galling compared with untreated control, fluopyram alone, or oxamyl (Figure 2). Galling was reduced 59 and 72% by 1,3-D and 1,3-D with fluopyram, respectively, relative to untreated in 2019. Soil population densities of *M. incognita* followed a similar numeric trend to galling in 2019, but there were no significant treatment effects (Figure 2). In 2020, oxamyl or 1,3-D with or without fluopyram reduced galling compared with fluopyram alone or untreated control (Figure 2). In 2020, galling reductions relative to untreated control were 88, 94, and 97% for oxamyl; 1,3-D; and 1,3-D plus fluopyram, respectively. Trends in 2020 *M. incognita* soil abundances were similar, with oxamyl or treatments with 1,3-D reducing soil abundances 95–98% relative to untreated, except that fluopyram alone was intermediate to untreated control and oxamyl (Figure 2). In both 2019 and 2020, nematicides had negative impacts on non-target, free-living nematodes, although impacts of specific chemistries varied somewhat by year. In 2019, any nematicide treatment reduced free-living nematode soil abundances 51–63% relative to untreated control, except that oxamyl alone was intermediate between untreated and the other nematicides (Figure 3). In 2020, oxamyl, 1,3-D, or 1,3-D with fluopyram reduced free-living nematode abundances 48–60% relative to untreated or fluopyram alone.

### Yield in 2019 and 2020 trials

**Figure 4:**
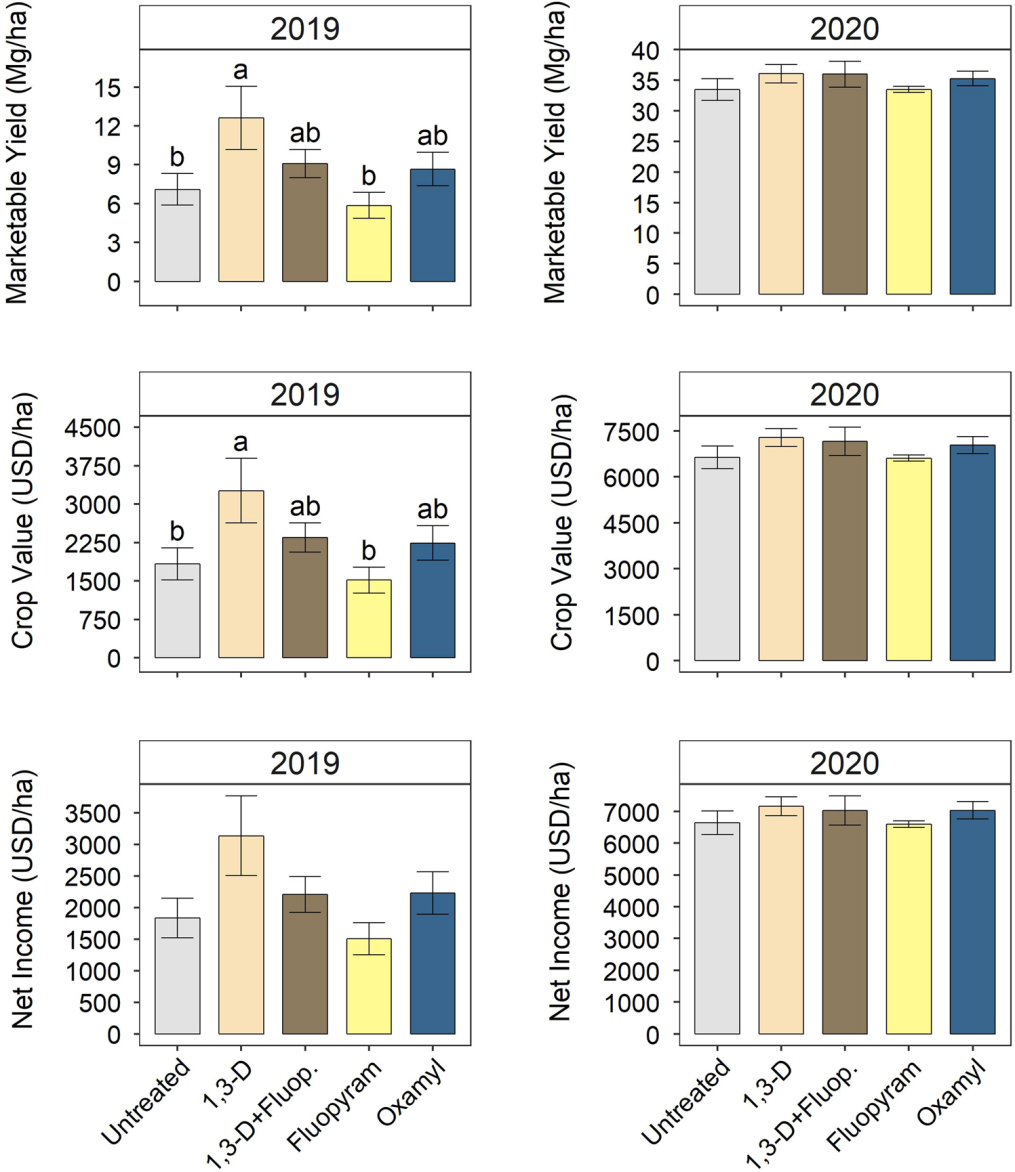
Nematicide effects on marketable yield, crop value, and net income in 2019 and 2020 trials. USD is United States dollars. Net income is crop value minus nematicide product cost, not including any labor or equipment costs. Within each subfigure, means with different letters are significantly different (*P * < * *0.05) based on Fisher’s protected LSD.

Nematicides affected crop performance in 2019, but not 2020. In 2019, marketable yield (Figure 4), small size category yield, total yield (Table 3), and crop value (Figure 4) were greater for 1,3-D alone than untreated or fluopyram alone. For 1,3-D alone, marketable yield, crop value, and small size yield increases were 78, 78, and 65%, respectively. However, net income in 2019 was not affected by nematicide treatments (Figure 4). In 2020, marketable (Figure 4), total, small, medium, and large yields (Table 3) were not significantly affected by nematicide treatments. Similarly, neither crop value nor net income was affected by nematicide treatments in 2020 (Figure 4).

**Table 3.** Tomato yield (Mg/ha) in different size categories as affected by nematicide treatments in 2019 and 2020.

Treatment	Cull^a^	Small^b^	Medium	Large	Total
	2019				
Untreated control	4.3	4.2a	2.2	0.7	11.4b
1,3-D	6.6	7.0b	4.2	1.4	19.2a
1,3-D+Fluopyram	5.4	5.2ab	2.9	1.0	14.4ab
Fluopyram	4.6	3.7b	1.6	0.6	10.5b
Oxamyl	6.1	5.4ab	2.4	0.8	14.8ab
	2020				
Untreated control	11.6	15.1	12.2	6.1	45.0
1,3-D	12.3	15.0	12.9	8.2	48.4
1,3-D+Fluopyram	12.3	16.5	12.5	7.0	48.3
Fluopyram	12.7	16.1	11.5	5.9	46.2
Oxamyl	12.7	15.8	12.4	7.1	47.9

Notes: ^a^Size diameter (cm) classes for cull, small, medium, and large, respectively, were: < 5.7, 5.7 to 6.3, 6.4 to 7.0, and > 7.0. ^b^Treatments with the same letters within the same column and year are not significantly different (Fisher’s protected LSD, *α* = 0.05). Columns with no letters have no significant nematicide effects (ANOVA, *α* = 0.05).

## Discussion

Fumigation with 1,3-D was the most consistent option for managing SRKN populations and symptoms, providing control of galling or soil abundances in all three trials. Oxamyl and fluopyram could provide control of SRKN, but not consistently from year to year. Oxamyl provided slightly better SRKN management than fluopyram, albeit in only one of the two years they were compared. Fumigation with 1,3-D alone was sufficient for SRKN control and supplementing with fluopyram did not add any significant benefits for managing SRKN. It was unclear why performance of the non-fumigant nematicides in managing SRKN soil abundances or root galling were better in a particular year than another. Factors such as temperature, soil moisture, and nematode pressure, may influence nematicide efficacy (Bao et al., 2013; Ou, 1998; Wheeler and Kaufman, 2003), but these factors did not provide a clear explanation for variation in non-fumigant nematicide efficacy at managing SRKN populations in this study. Soil moisture was monitored and regulated by irrigation and soil temperatures were similarly warm (27–32°C) during nematicide application each year. Nematode pressure from SRKN was high enough to detect any treatment effects each year, based on final SRKN soil abundances and root gall ratings. Excess nematode pressure in a particular year, which could overwhelm nematicide control, also did not seem to be a factor as control by non-fumigants was perhaps worst in 2019 when SRKN soil abundances and gall ratings were somewhat lower.

Yield responses and total crop value generally corresponded to SRKN population management, aside from 2020, with 1,3-D again providing the best response. While there were no statistical differences in net income, numeric trends clearly followed yield and crop value and suggest that applying 1,3-D was the most economically advantageous option. The non-fumigant nematicides oxamyl and fluopyram provided some value for increasing yield, with fluopyram providing equal yield to 1,3-D in the preliminary trial, just not as consistently across years as 1,3-D. As with SRKN population management, there was no yield advantage in supplementing fluopyram if 1,3-D had already been applied.

There was no yield response in 2020 despite similar or greater nematode pressure-based on similar galling ratings and numerically increased final SRKN soil abundances-compared with 2018 and 2019, when there were yield responses. A possible explanation is that tomato tolerance to SRKN infection was improved in 2020 because conditions were more favorable for tomato growth. Yields in 2020 were elevated in comparison to 2019, due largely to heavy whitefly (Aleyrodidae) infestation and subsequent Tomato Leaf Curl Yellow Virus infection in 2019, whereas insect infection was minimal in 2020. Total yields were comparable in 2020 and 2018, but there more pickings in 2018 and plant vigor was subjectively better in 2020.

Results of this study were generally consistent with prior research in related cropping systems. Fumigation using 1,3-D is known to be among the best and most consistent current options for nematode management in high value crops (Desaeger and Csinos, 2006; Desaeger et al., 2017; Hamill and Dickson, 2005; Watson and Desaeger, 2019). The inconsistent efficacy of oxamyl in this study is similar to past research. Oxamyl as a standalone nematicide has ranged from consistently effective (Desaeger and Csinos, 2006) to inconsistent based on year or location within a given study (Desaeger et al., 2004; Khanal and Desaeger, 2020) to ineffective (Desaeger et al., 2017; Hamill and Dickson, 2005). Prior fluopyram field tests against *Meloidogyne* spp. in plasticulture vegetable production, representing similar conditions to this study, have typically shown inconsistent efficacy (Desaeger and Watson, 2019; Khanal and Desaeger, 2020; Watson and Desaeger, 2019), although fluopyram was consistently effective in one report on tomato production (Ji et al., 2019).

Direct comparisons of fluopyram to oxamyl or 1,3-D have been limited in number. In microplot tests, fluopyram was either better (Hajihassani et al., 2019) or worse (Jones et al., 2017) than oxamyl for SRKN management. In field tests, fluopyram and oxamyl have performed similarly (Desaeger and Watson, 2019; Khanal and Desaeger, 2020). A mixture of 1,3-D and chloropicrin performed better than fluopyram and supplementing fumigation with fluopyram did not improve management in strawberry production (Watson and Desaeger, 2019). Most prior research in plasticulture on supplementing fumigation with non-fumigant nematicides has focused on carbamate nematicides, such as oxamyl, and results have been mixed. Applying oxamyl in addition to 1,3-D or 1,3-D with chloropicrin has either had no benefit (Desaeger et al., 2017; Hamill and Dickson, 2005), marginal benefits (Desaeger and Csinos, 2006) or consistent benefits (Desaeger et al., 2004) relative to fumigation alone.

In this study, free-living nematodes were either less sensitive to nematicides or rebounded more quickly than SRKN as SRKN soil abundances were reduced by up to 98% relative to untreated whereas free-living nematode soil abundances reductions ranged from 48 to 63%. In addition to being the most effective against target nematodes, 1,3-D also had the most consistent, negative impact on free-living nematodes in this study. This is consistent with prior research showing 1,3-D negatively impacts a range of non-target, free-living nematodes (Sanchez-Moreno et al., 2010; Timper et al., 2012; Watson and Desaeger, 2019). Fluopyram and oxamyl also had negative impacts on free-living nematodes, but were not as consistent, with lesser impacts on free-living nematodes roughly corresponding to lesser efficacy against SRKN. Because all products tested had similar non-target effects when also effective against SRKN, none of them could be practically recommended as an effective nematode control option that minimizes non-target effects based on this study. However, there could be differences among nematicides in impacts on different trophic groups, ecological niches, or taxonomic units (Grabau and Chen, 2016; Grabau et al., 2020; Watson and Desaeger, 2019) that were not detected in this study as it focused on total free-living nematodes only. Study results on oxamyl and fluopyram are relatively consistent with past research, albeit a limited number of studies. Oxamyl has had negative impacts on free-living nematodes while also being effective against target RKN (Carrascosa et al., 2015; Ntalli et al., 2018). Fluopyram has been more variable in non-target effects ranging from minimal (Grabau et al., 2020; Watson and Desaeger, 2019) to extensive (Waldo et al., 2019).

In summary, 1,3-D alone was the most efficient option among the nematicides tested. While the non-fumigants oxamyl and fluopyram were not as consistent as fumigation with 1,3-D, they could provide SRKN management and may be valuable tools when fumigation is not feasible. Supplementing 1,3-D fumigation with fluopyram nematicide application does not seem to improve SRKN management or production beyond fumigation alone. Non-target effects on free-living nematodes should not influence nematicide choice among 1,3-D, oxamyl, or fluopyram as each nematicide had similar deleterious non-target effects when effective against SRKN.
